# Clinical impact of functional CYP2C19 and CYP2D6 gene variants on treatment outcomes in patients with depression: a Danish cohort study

**DOI:** 10.1192/j.eurpsy.2022.591

**Published:** 2022-09-01

**Authors:** L. Thiele, K. Ishtiak-Ahmed, J. Thirstrup, C. Lunenburg, D. Müller, C. Gasse

**Affiliations:** 1 Aarhus University Hospital Psychiatry, Department Of Affective Disorders, Aarhus N, Denmark; 2 Aarhus University, Department Of Clinical Medicine, Aarhus, Denmark; 3 Aarhus University, Department Of Clinical Medicine, Aarhus N, Denmark; 4 Institute of Medical Science, University of Toronto, Department Of Psychiatry, Toronto, Canada; 5 Centre for Addiction and Mental Health, Pharmacogenetics Research Clinic, Toronto, Canada

**Keywords:** pharmacogenetics, sertraline, (es)citalopram and fluoxetine, Depression

## Abstract

**Introduction:**

Pharmacogenetic (PGx) targets to optimize drug therapy, but its implementation is rare.

**Objectives:**

We evaluate the clinical utility of PGx testing in psychiatry by investigating the one-year risks of clinical outcomes in patients with depression taking sertraline, (es)citalopram or fluoxetine by their Cytochrome P450 (CYP) 2C19/2D6 phenotypes.

**Methods:**

We investigated 17,297 individuals born between 1981-2005 with a depression diagnosis between 1996-2012 from the iPsych2012 case-cohort. Based on array-based single-nucleotide-polymorphism genotype data, individuals were phenotyped as CYP2C19/CYP2D6 normal (NM, reference group), ultra-rapid- (UM), rapid- (RM), intermediate- (IM), or poor-metabolizer (PM). Outcomes were treatment switching or discontinuation, psychiatric in-, out-, and emergency room contacts (ER), and suicide attempt/self-harm. Incidence rate ratios (IRR) by age groups were estimated using Poisson regression analysis with 95% confidence intervals, adjusted for potential confounders.

**Results:**

Risks of switching (IRR=1.89[1.22-2.93]), ERs (1.69 [1.01-2.81]) and suicide attempt/self-harm (2.73 [1.49-5.01]) were higher in CYP2C19 PMs <19 years taking (es)citalopram. Fluoxetine users <19 years had a decreased risk of discontinuation in CYP2D6 PMs (0.5 [0.27-0.95]) and decreased risk of out-patient contacts in CYP2D6 PMs and IMs (IRR_IM_=0.83 [0.68-1.00] and IRR_PM_=0.59 [ 0.37-0.96]). We observed an increased risk for ERs in CYP2D6 PMs aged 19-25 years taking fluoxetine (4.53 [1.54-13.35]). In CYP2C19 UMs >25 years taking (es)citalopram the risk of suicide attempt/self-harm was more than three-fold increased (3.64 [ 1.01-13.19]). We found no significant results in users of sertraline.

**Conclusions:**

PGx variability was associated with treatment outcomes in depression in patients with CYP2C19 PM or UM status taking (es)citalopram, or CYP2D6 PM or IM status taking fluoxetine.

**Disclosure:**

No significant relationships.

